# Therapeutic of Candesartan and Music Therapy in Diabetic Retinopathy with Depression in Rats

**DOI:** 10.1155/2021/5570356

**Published:** 2021-03-26

**Authors:** Chengping Luo, Huimin Fan, Shaojun Li, Yuling Zou

**Affiliations:** ^1^Music College of Jiangxi Normal University, Nanchang 330022, China; ^2^Department of Ophthalmology, The Second Affiliated Hospital of Nanchang University, Nanchang 330006, China

## Abstract

This study aimed to investigate the therapeutic effects of candesartan combined with music therapy on diabetic retinopathy with depression and to assess the molecular mechanisms. Associated animal model of diabetes mellitus and depression was established in rats. Pathological changes in the hippocampus were detected by haematoxylin eosin (H&E) staining. Terminal deoxynucleotidyl transferase dUTP nick-end labeling (TUNEL) was used to detect retinal cell apoptosis. Angiotensin II (Ang II) in peripheral blood and neurotransmitters, including serotonin (5-HT), dopamine (DA), and norepinephrine (NE) in the hippocampus, was measured by enzyme linked immunosorbent assay (ELISA). Fluorescence quantitative PCR and western blotting were used to detect the expression of brain-derived neurotrophic factor (BDNF) and c-fos in the hippocampus. Our data showed that chromatin aggregation and cytoplasmic vacuolation were observable in the hippocampal cells of the rats in the model group, while candesartan and music therapy could reduce morphological changes in the hippocampus of diabetic rats with depression. Compared with the control group, the apoptosis of retinal cells was significantly higher, the contents of 5-HT, DA, and NE in the hippocampus were significantly lower, Ang II level in peripheral blood was significantly higher, and the expression of BDNF and c-fos in the hippocampus decreased significantly in the model group. By contrast, candesartan or candesartan + music therapy ameliorated the changes in retina cell apoptosis, reduction of neurotransmitters, increase in AII, and the expression of c-fos and BDNF. Especially, music therapy further improved the effects of candesartan on retina cell apoptosis and neurotransmitter release in diabetic retinopathy rats with depression. In conclusion, candesartan and music therapy have an additive effect in DM with both visual impairment and depression, which might serve a potential alternative treatment for this complex disease.

## 1. Introduction

Diabetes mellitus (DM) is a type of syndrome characterized by persistent hyperglycemia, which is caused by insulin deficiency or insulin resistance with an unknown etiology and pathogenesis. As a chronic long-term disease, diabetes patients are required to control sugar intake, monitor blood glucose, and have a long-term medication or insulin injection, which greatly influence the quality of life [1]. The occurrence of various complications may also threaten patients, which will inevitably lead to fear, pessimism, and anxiety [2]. Depression is a kind of emotional disorder characterized by sadness, disappointment, decreased activity ability, slow thinking, and cognitive dysfunction [3]. The incidence of depression in patients with diabetes is higher than normal population as the hyperglycemia likely damages neurons or neural circuit related to depression [4]. Hyperglycemia could also damage visual function, leading to retinopathy [5]. Moreover, individuals with both diabetes and visual impairment are prone to experience depressive symptoms [6, 7]. Meanwhile, depression also has complex adverse effects on their physiological function, metabolic regulation, and the occurrence and development of complications of diabetes [8]. Therefore, it is of particular importance to find treatment for DM with both visual impairment and depression.

Candesartan is a new type of antagonist of angiotensin II (Ang II) receptor. It plays a pharmacological role through noncompetitive combination with type I of Ang II receptor with the characteristics of strong and long-term effect, as well as high selectivity. After oral administration, candesartan is rapidly absorbed through gastrointestinal tracts [9]. Candesartan could repair the organ damages in other pathological parts, including left ventricular hypertrophy and thickening of the inner middle membrane. Recently, candesartan has been reported to prevent retinopathy [10].

Music therapy, as a nondrug assisted intervention, has a good effect on reducing pain and anxiety, enhancing patients' comfort and promoting recovery [11]. However, the associated function of candesartan and music therapy in the animal model of DM with both visual impairment and depression is not validated. Therefore, this study established a model of DM with depression in Sprague Dawley (SD) rats and evaluated the effects of candesartan combined with music therapy on hippocampal pathological changes, retinal cell apoptosis, neurotransmitters, and cell activations in DM with depression. This study would provide new choice for the treatment of DM with both visual impairment and depression.

## 2. Materials and Methods

### 2.1. Animals and Models

44 male SD rats (250–300 g) were purchased from Hunan Shrek Jingda Experimental Animal Co., Ltd. (scxk (Hunan) 2016–0002). After a 7-day adaption, the rats were fasted for 12 h followed by a single intraperitoneal injection of 60 mg/kg streptozotocin (STZ) to produce DM. 72 h later, the blood glucose level was detected, and the rats with blood glucose value >16.7 mM were identified as diabetic rats. In control rats, similar volume saline was injected. Diabetic rats were raised separately and received 7 different kinds of stress stimulations, including cold water swimming (10°C, 5 min), tail clamping (3 min), water deprivation (24 h), fasting (24 h), inclined cage (24 h), day-night inversion (24 h), and environment with high humidity (24 h) for 28 days. One kind of stimulation was randomly selected each day. The rats in the candesartan group were orally given candesartan (10 mg/kg/d) 1 h before stimulation for 28 days. The rats in the candesartan + music treatment group received candesartan treatment (10 mg/kg/d) and music treatment 1 h before stimulation for 28 days. After stress, the rats received candesartan (10 mg/kg/d) or candesartan + music treatment for another 28 days. Experimental groups were divided into four groups (*N* = 5 in each group): a normal control group (control), a model group, a candesartan (10 mg/kg/d) group, and a candesartan (10 mg/kg/d) + music therapy (candesartan + music) group. The rats in the candesartan + music therapy group received 1 h music every day for 8 weeks. Mozart's double Piano Sonata in D major K448 was selected based on its function in stimulating brain activity [12]. Thereafter, the peripheral blood, retina tissue, and hippocampus were collected for the subsequent experiments.

### 2.2. HE Staining

The hippocampal tissues were fixed in 4% paraformaldehyde overnight at 4°C. The tissues were taken out and washed with running water for several hours and then dehydrated with 70%, 80%, and 90% ethanol, alcohol and xylene for 15 min, xylene I for 15 min, and xylene II for 15 min. The tissues were put back in the mixture of xylene and paraffin for 15 min and then in paraffin I and paraffin II for 50–60 min, respectively. The tissues underwent paraffin embedding and were sectioned into 10 *μ*m thickness slides. The paraffin sections were baked, dewaxed, and hydrated. The sections were immersed into the aqueous solution of haematoxylin for 3 min and eosin for 3 min at room temperature in the dark. The slides were sealed and imaged under a light microscope with the magnification of 200X.

### 2.3. TUNEL Assay

The hippocampal slices were prepared as above statement and placed in the oven at 65°C for 2 h; the slices were placed in xylene for 10 min; the slices were placed in 100% ethanol, 100% ethanol, 95% ethanol, 80% ethanol, and dH_2_O for 5 min, respectively. The slices were transferred into a wet box, 50 *μ*g/ml of proteinase K was added to each sample, and the reaction lasted for 30 min at 37°C. TUNEL detection solution (C1088, Beyotime, Ningbo, China) was added to each slide and incubated at 45°C in the dark for 2 h. The slides were washed with PBS for three times (each time for 5 min). The slide was sealed with mounting solution and observed under fluorescence microscope as previously described [13].

### 2.4. ELISA

Ang II level in peripheral blood and neurotransmitters including serotonin (5-HT), dopamine (DA), and norepinephrine (NE) in the hippocampus was detected using the ELISA following the instructions of the kits (Ang II: MM-0211R1; 5-HT: MM-0442R1; DA: MM-0355R1; NE: MM-20302R1, MMBIO, Nanjing, China) as previously described [14]. The absorbance (OD value) of each well was measured at the wavelength of 450 nm within 15 min.

### 2.5. Fluorescence Quantitative PCR

Total RNA was extracted from the hippocampal tissues using the Trizol Reagent (CW0580S, CWBIO), and then, cDNA was synthesized according to the reverse transcription kit (CW2569M, CWBIO). The expression of BDNF and c-fos was calculated by using cDNA as template and fluorescence quantitative PCR. The primers are listed in Table 1.

### 2.6. Western Blotting

The protein was extracted from the hippocampal tissues for western blot analysis. After determining the protein concentration using the BCA method, the proteins were processed in 12% sodium dodecylsulphate polyacrylamide gel electrophoresis. After transferring onto the PVDF membrane, the nonspecific staining was blocked by 5% defat milk. The membrane was incubated with the antibodies, including BDNF (1 : 1000, DF6387, Affinity), c-fos (1 : 1000, ab222699, Abcam), and *β*-actin (1 : 3000, ab8227, Abcam). Thereafter, the membranes were probed with anti-mouse immunoglobulin (Ig) G or anti-rabbit IgG horseradish peroxidase-conjugated secondary antibodies at room temperature for 2 h.

### 2.7. Statistical Analyses

All data were expressed at mean and standard deviation and analyzed by SPSS 19.0, and the significant difference was analyzed by one-way ANOVA following with Newman–Keuls as the post hoc test. A *P* < 0.05 was considered to be a significant difference.

## 3. Results

### 3.1. Behavioral Changes in the Animals

**Figure 1:**
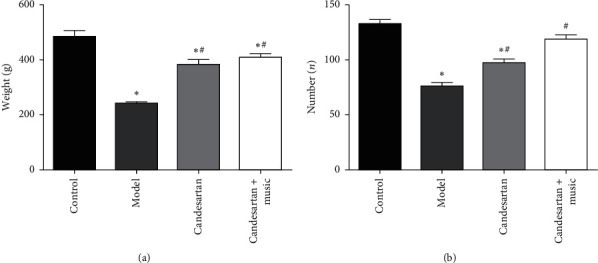
Behavioral changes in the animals: (a) weight of the rats; (b) exercise frequency of the rats. Compared with the control group, ^*∗*^*P* < 0.05; compared with the model group, ^#^*P* < 0.05 (*N* = 5 in each group).

Compared with the control group, the weight and exercise frequency of the rats in the model group were significantly decreased (*P* < 0.05). By contrast, the weight and exercise frequency of rats in the candesartan + music therapy group were significantly increased compared with the model group (*P* < 0.05) (Figure 1). These data suggest that candesartan and music therapy might be effective in the treatment of DM with both visual impairment and depression.

### 3.2. Pathological Changes in Hippocampal Tissues

**Figure 2:**
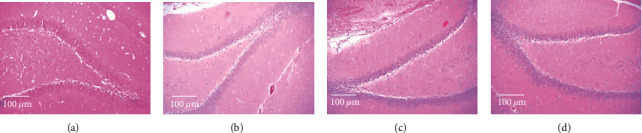
Pathological changes in the hippocampal tissues. Candesartan and music therapy ameliorated the pathological changes in the hippocampus: (a) control; (b) model; (c) candesartan; (d) candesartan + music.

As shown in Figure 2, the hippocampal cells of the control group were normally arranged. In the model group, deep staining and vacuolar degeneration were found in the hippocampal cells. The number of vacuolar cells in the hippocampus of the candesartan group and candesartan + music therapy group remarkably decreased. These data indicate that candesartan and music therapy could repair hippocampal injury in the model of DM with both visual impairment and depression.

### 3.3. Candesartan and Music Therapy Reduced the Retina Cell Apoptosis

**Figure 3:**
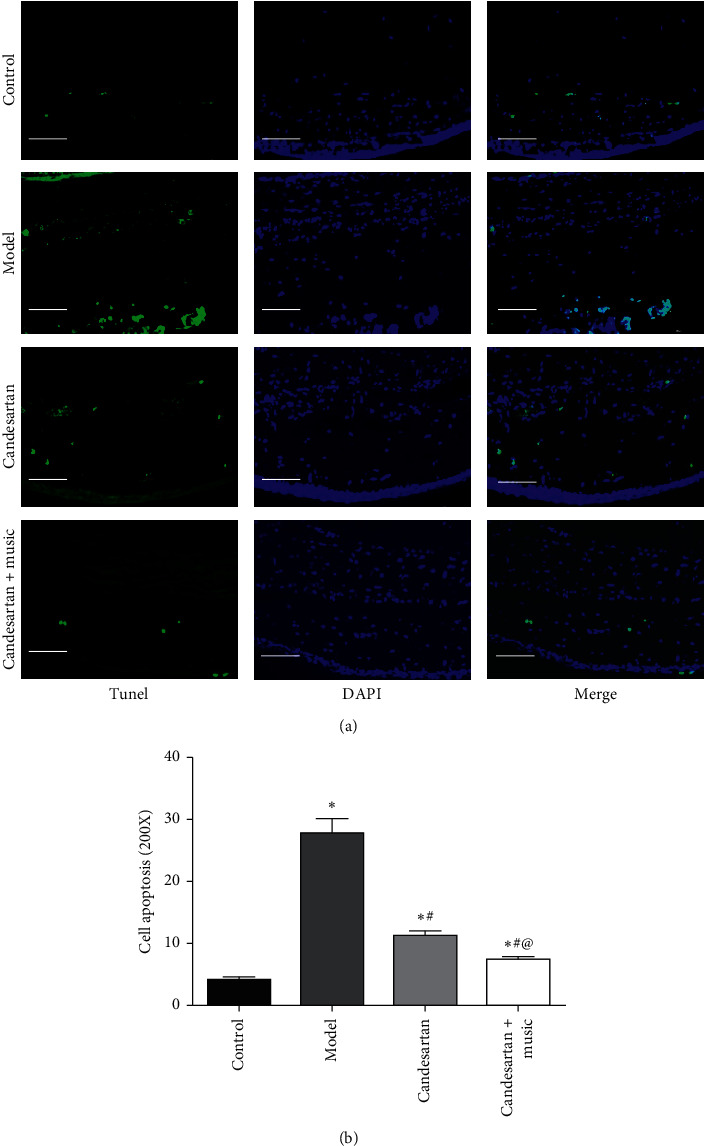
Candesartan and music therapy reduced the retina cell apoptosis. Compared with the control group, ^*∗*^*P* < 0.05; compared with the model group, ^#^*P* < 0.05; compared with the candesartan group, ^@^*P* < 0.05 (*N* = 5 in each group).

Compared with the control group, the apoptotic cells of retina tissue in the model group were significantly higher (*P* < 0.05); compared with the model group, the apoptotic cells of retina tissue in the candesartan group and candesartan + music therapy group was significantly lower (vs. model, *P* < 0.05). Interestingly, music therapy further improved the effects of candesartan on retina cell apoptosis (vs. candesartan, *P* < 0.05) (Figure 3).

### 3.4. Candesartan and Music Therapy Elevated Hippocampal 5-HT, DA, and NE

**Figure 4:**
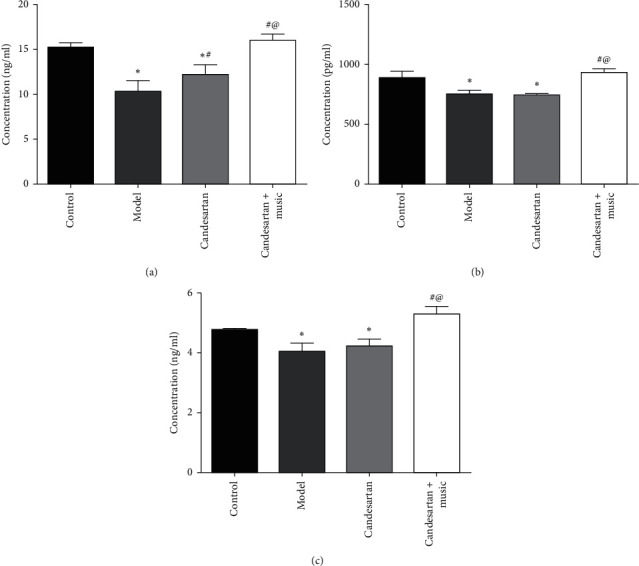
Candesartan and music therapy elevated hippocampal 5-HT (a), DA (b), and NE (c). Compared with the control group, ^*∗*^*P* < 0.05; compared with the model group, ^#^*P* < 0.05; compared with the candesartan group, ^@^*P* < 0.05 (*N* = 5 in each group).

As shown in Figure 4, compared with the control group, 5-HT, DA, and NE levels in the hippocampus of the model group decreased significantly (*P* < 0.05); compared with the model group, 5-HT, DA, and NE levels in the hippocampus of candesartan + music therapy group increased significantly (*P* < 0.05). Compared with candesartan treatment, candesartan + music therapy had a strong effect on hippocampal 5-HT, DA, and NE levels (vs. candesartan, *P* < 0.05).

### 3.5. Candesartan and Music Therapy Reduced AII Level in Peripheral Blood

**Figure 5:**
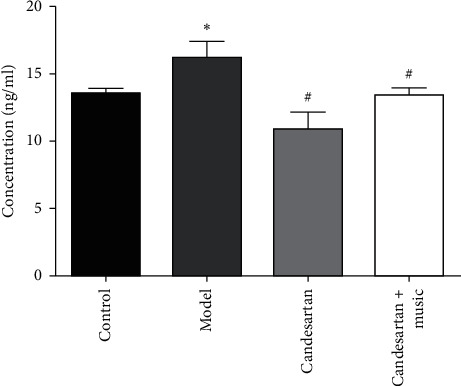
Candesartan and music therapy reduced AII level in peripheral blood. Compared with the control group, ^*∗*^*P* < 0.05; compared with the model group, ^#^*P* < 0.05 (*N* = 5 in each group).

Compared with the control group, Ang II in the peripheral blood of the model group increased significantly (*P* < 0.05); compared with the model group, Ang II in the peripheral blood of the candesartan group and candesartan + music therapy group decreased significantly (*P* < 0.05) (Figure 5).

### 3.6. Candesartan and Music Therapy Promoted BDNF and c-fos in the Hippocampus

**Figure 6:**
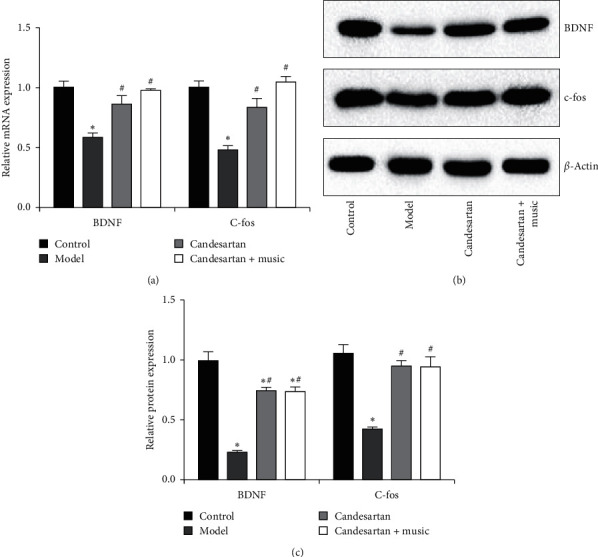
Candesartan and music therapy promoted BDNF and c-fos in the hippocampus: (a) mRNA expression; (b) protein expression. Compared with the control group, ^*∗*^*P* < 0.05; compared with the model group, ^#^*P* < 0.05 (*N* = 5 in each group).

Compared with the control group, BDNF and c-fos expression in the hippocampus of model group decreased significantly (*P* < 0.05); compared with the model group, BDNF and c-fos expression in the hippocampus of candesartan + music therapy group increased significantly (*P* < 0.05) (Figure 6).

## 4. Discussion

In this study, we evaluated the effects of candesartan combined with music therapy in the treatment of diabetic retinopathy with depression. Candesartan and music therapy prohibit hippocampal injury, reduce peripheral AII and retina cell apoptosis, and promote the release of neurotransmitters to delay diabetes mellitus with depression. Interestingly, music therapy further improved the effects of candesartan on retinal cell apoptosis and neurotransmitter release in diabetic retinopathy with depressive rats. This study would reveal a potential treatment of diabetic retinopathy with depression.

DM associated with depression seriously affects the psychosomatic health, social interaction, and physical activity of patients, which directly affects the quality of life of patients. Depressive patients usually suffer from emotional loss, loss of interest and pleasure, and lack of concentration or fatigue [15]. Candesartan is a new type of oral angiotensin receptor blockers (ARBs) used in clinic because it selectively blocks the combination of Ang II and AT1R, thus blocking the biological effect of Ang II [16]. Some studies have shown that candesartan can reduce the loss of peripheral cells, thus delaying the development of diabetic retinopathy [17–19]. In this study, we demonstrated that candesartan also reduced Ang II in peripheral blood, which might contribute to its protection against retina cell apoptosis in the diabetic retinopathy with depression model.

Music therapy can relieve anxiety and pain [20, 21]. It uses the physical function of sound waves to act on human sensory organs through the melody, speed, and tone of musical instruments and directly produces resonance effect on organs in the body [22]. When the vibration produced by music resonates with the internal organs, it can play a role through complex neurohumoral regulation, reduce the release of stress hormones such as cortisol, and affect the release of endorphin [23]. In this study, the pathological results showed that different degrees of deep staining and vacuolation injury were observable in the hippocampal tissue after the establishment of diabetes mellitus with depression model, and the apoptosis of retinal tissue was significantly increased; after treatment with candesartan and music, the number of vacuolation cells in the hippocampus was reduced, and the apoptosis of retinal tissue was also reduced. These results indicate that candesartan and music therapy have a certain effect on diabetic depression model.

Ang II is the most biologically active substance, and its multiple biological effects may be involved in the development of diabetic retinopathy [24]. Ang II can cause the contraction of tissue arterioles and microvessels, weaken the sensitivity of tissue to insulin, inhibit the fibrinolysis system, promote the expression of a variety of other cytokines and growth factors through autocrine and paracrine mechanisms, aggravate inflammatory response, increase vascular permeability, and induce apoptosis [25]. Ang II participates in peripheral cell damage in diabetic retinopathy, and the degree of damage is positively related to the level of Ang II [26]. Candesartan can delay the development of diabetic retinopathy by inhibiting the production of Ang II and reducing the loss of peripheral cells [27]. The results of present study also demonstrated that Ang II level in the peripheral blood of model group increased, while that of Ang II decreased after treatment with candesartan, which was consistent with the above results.

Depression is related to the change in neurotransmitters [28]. DA is a catecholamine neurotransmitter and plays a key role in cognitive function [29]. When a large number of neurons are lost, patients will show a variety of cognitive disorders [30]; NE also participates in cognitive-related processes such as learning and memory, execution process, and attention regulation. When NE neurons are destroyed, NE level is also decreased [31]; 5-HT is considered to be an important monoamine neurotransmitter for mood regulation and learning and memory [32]. In this study, the content of neurotransmitter DA, NE, and 5-HT decreased significantly after the establishment of diabetes mellitus with depression, and the content of neurotransmitter DA, NE, and 5-HT increased after music therapy. These results indicate that depression in DM is related to the decrease in these neurotransmitters. Music therapy can increase these neurotransmitters so as to achieve a certain effect.

C-fos participates in brain cell apoptosis in the process of gene expression upon stimulation [33]. BDNF is an important member of neurotrophic factor family [34]. BDNF could promote cell differentiation and proliferation of neural stem cells, has nutrition effects on many types of neurons, has a greater impact on the plasticity of neurons and the synthesis of neurotransmitters, and is also related to learning and memory [35]. In addition, other studies have shown that BDNF plays an important role in the pathogenesis of severe depression [34, 36, 37]. BDNF is involved in the survival of neurons and the regeneration of dopamine, 5-HT, and cholinergic neurons in the central nervous system, and it is also a molecular marker of synaptic plasticity [38]. Exogenous BDNF has antidepressant effect in the animal model of depression [37]. BDNF can resist stress-induced neuronal damage and affect the regeneration of neurons in the hippocampus, which is considered to be related to the pathogenesis of emotional disorders [39]. Our present study showed that the expression of c-fos and BDNF decreased after the establishment of diabetic depression model, and the expression of c-fos and BDNF increased after candesartan and music therapy. These results suggest that candesartan and music may play a role in the treatment of diabetic depression by activating the expression of c-fos and BDNF.

There were still some limitations. First, more behavioral tests should be performed to detect the depressive-like behaviors. Second, the direct association between DM and depression has not been validated in this study. If possible, the common mechanisms for DM and depression should be investigated. Additionally, the effects of candesartan and music therapy on the level of blood glucose should also be investigated.

In conclusion, candesartan and music therapy prohibit hippocampal injury, reduce peripheral AII and retina cell apoptosis, and promote the release of neurotransmitters to delay DM with depression. Candesartan and music therapy might serve a potential alternative treatment for this complex disease.

## Figures and Tables

**Table 1:** Primer sequences of the genes.

Genes	Primer sequences	Sequence length (bp)	Product length (bp)	Annealing temperature (°C)
*BDNF F*	GATCCACTGAGCAAAGCCGA	20	137	59.8
*BDNF R*	CTCACCTGGTGGAACATTGTG	21		

*c-fos F*	CCAAGCGGAGACAGATCAAC	20	138	58.8
*c-fos R*	CCCAGGTCATTGGGGATCTT	20		

*β-Actin F*	ACGGTCAGGTCATCACTATC	20	90	56.5
*β-Actin R*	TGCCACAGGATTCCATACC	19		

## Data Availability

The datasets used and/or analyzed during the current study are available from the corresponding author on reasonable request.
